# Nonsense‐mediated decay factor SMG7 sensitizes cells to TNFα‐induced apoptosis via CYLD tumor suppressor and the noncoding oncogene *Pvt1*

**DOI:** 10.1002/1878-0261.12754

**Published:** 2020-07-13

**Authors:** Limeng Yang, Vanessa A. N. Kraft, Susanne Pfeiffer, Juliane Merl‐Pham, Xuanwen Bao, Yu An, Stefanie M. Hauck, Joel A. Schick

**Affiliations:** ^1^ Genetics and Cellular Engineering Group Institute of Molecular Toxicology and Pharmacology Helmholtz Zentrum Munich GmbH German Research Center for Environmental Health Neuherberg Germany; ^2^ Research Unit Protein Science Helmholtz Zentrum Munich GmbH German Research Center for Environmental Health Neuherberg Germany; ^3^ Institute of Radiation Biology Helmholtz Zentrum Munich GmbH German Research Center for Environmental Health Neuherberg Germany; ^4^ Department of Chinese Medicine National Cancer Center/National Clinical Research Center for Cancer/Cancer Hospital Chinese Academy of Medical Sciences and Peking Union Medical College Beijing China

**Keywords:** apoptosis, cancer, CYLD, lncRNA, nonsense‐mediated decay, *Smg7*

## Abstract

Nonsense‐mediated decay (NMD) proteins are responsible for the surveillance and degradation of aberrant RNAs. Suppressor with morphogenetic effect on genitalia 7 (SMG7) is an NMD complex protein and a regulator of tumor necrosis factor (TNF)‐induced extrinsic apoptosis; however, this unique function has not been explored in detail. In this study, we show that loss of *Smg7* leads to unrestricted expression of long noncoding RNAs (lncRNAs) in addition to NMD targets. Functional analysis of *Smg7^−/−^* cells showed downregulation of the tumor suppressor cylindromatosis (CYLD) and diminished caspase activity, thereby switching cells to nuclear factor‐κB (NF‐κB)‐mediated protection. This positive relationship between *SMG7* and *CYLD* was found to be widely conserved in human cancer cell lines and renal carcinoma samples from The Cancer Genome Atlas. In addition to CYLD suppression, upregulation of lncRNAs *Pvt1* and *Adapt33* rendered cells resistant to TNF, while pharmacologic inhibition of NF‐κB in *Pvt1‐*overexpressing TNF‐resistant cells and *Smg7*‐deficient spheroids re‐established TNF‐induced lethality. Thus, loss of SMG7 decouples regulation of two separate oncogenic factors with cumulative downstream effects on the NF‐κB pathway. The data highlight a novel and specific regulation of oncogenic factors by SMG7 and pinpoint a composite tumor suppressor role in response to TNF.

## Graphical abstract

Yang *et al*. found that mutation of the nonsense‐mediated decay factor SMG7 enhanced resistance to tumor necrosis factor (TNF)‐induced apoptosis via two oncogenic pathways. Downregulation of CYLD and overexpression of the long noncoding RNA *Pvt1* converge to promote NF‐κB activation. The results indicate a composite tumor suppressor role for SMG7 in response to TNF.

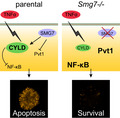

**Abbreviations**
BF: brightfield
CCLE: Cancer Cell Line Encyclopedia
CRISPRa: CRISPR activation
CRISPRi: CRISPR inhibition
DISC: death‐inducing signaling complex
GSEA: gene set enrichment analysis
IFNγ: interferon gamma
KD: knockdown
KIRC: kidney renal clear cell carcinoma
lincRNA: long intergenic noncoding RNA
lncRNA: long noncoding RNA
LPS: lipopolysaccharide
MF: mouse fibroblasts
miRNA: micro‐RNA
NMD: nonsense‐mediated decay
onco‐lncRNA: oncogenic lncRNA
PI: propidium iodide
PTC: premature termination codon
RCC: renal cell carcinoma
siRNA: small interfering RNA
snRNA: small nuclear RNA
TCGA: The Cancer Genome Atlas
TNFα: tumor necrosis factor‐alpha
TRAIL: TNF‐related apoptosis‐inducing ligand
TSS: transcription start site
TWEAK: TNF‐related weak inducer of apoptosis
zVAD: Z‐VAD‐FMK

## 1 Introduction

Dysregulated gene expression is a hallmark of progressive cancers. As a result of cancer‐causing mutations, tumors upregulate unconventional transcripts such as noncoding, micro‐RNA, and pseudogenes. Many of these transcripts are recognized by the presence of a premature termination codon (PTC) and are targeted for degradation. Although PTC‐containing transcripts are tightly controlled in healthy untransformed cells, their dysregulation underlies many human diseases [1]. Up to 30% of all heritable cancers are thought to result from such nonsense‐mutations [2].

The major process for degrading aberrant transcripts is known as nonsense‐mediated decay (NMD). Surprisingly, more than 10% of mRNAs are susceptible to this phenomenon. These include PTCs and extended 3′ UTRs, as well as many others with yet undefined signatures [3]. An emerging class of NMD targets includes long noncoding RNAs (lncRNAs). LncRNAs are polymerase II transcripts that lack an open reading frame but have diverse regulatory roles as protein cofactors, micro‐RNA sponges, transcriptional enhancers, and antisense RNAs [4]. Due to this versatility, they play a wide‐ranging role in human cancer [5, 6]. It follows that onco‐lncRNAs like *ANRIL, GAS5,* and *MALAT1* are increasingly identified as tumor suppressors and oncogenes [4, 7], whereas others such as *HOTAIR* serve as functional biomarkers [8, 9].

SMG7 is an RNA surveillance factor that functions together with up‐frameshift (UPF) factors to deadenylate and degrade target RNAs [10, 11]. Several studies have highlighted SMG7 interaction with P53 as influencing cellular survival [12, 13]. SMG7 has also been identified in a large cohort to be associated with prostate cancer [14]. Our previous work identified *Smg7* in a whole‐genome mutagenesis screen against TNFα, a pleiotropic cytokine that can induce extrinsic apoptosis [15]. TNFα can induce cytotoxicity in tumors [16] but also plays a central role in NF‐κB activation and inflammation. Yet, the functional role of downstream targets of SMG7 with respect to TNFα and tumor biology is poorly understood.

In this study, we examined gene expression in *Smg7^−/−^* cells and found that lncRNAs rather than PTC‐containing transcripts were preferentially overexpressed, indicating that SMG7 uniquely targets this class of transcripts. Further evaluation of the TNFα pathway in these cells identified decreased CYLD tumor suppressor protein as the source of apoptosis resistance. CYLD is a negative regulator of NF‐κB that acts at the pathway branchpoint between apoptosis and NF‐κB activation. Accordingly, downregulation of CYLD in *Smg7^−/−^* cells reduced caspase activity and promoted NF‐κB‐mediated survival, while *Cyld* overexpression and NF‐κB pharmacological inhibition re‐established TNFα sensitivity. Strikingly, *CYLD* and *SMG7* expression showed a near‐universal correlation in diverse human cancer cell lines and clear cell renal cell carcinoma patient survival.

We further examined noncoding RNAs as preferred degradation targets of SMG7. Overexpression of two lncRNAs, *Pvt1* and *Adapt33,* showed robust protection against TNFα that increased further upon *Smg7* knockdown. *PVT1* is an oncogene identified in Burkitt's lymphoma [17], while *Adapt33* is a stress‐induced transcript upregulated in response to apoptotic stimuli [18]. Administration of TNFα to 3D spheroids produced widespread cell death in parental cells, while *Smg7^−/−^* spheroids showed compaction with viability. Nevertheless, pharmacological sensitization of the NF‐κB pathway in both cell lines suppressed CYLD‐ and *Pvt1*‐mediated survival. Taken together, the direct relationship with CYLD and regulation of oncogenic lncRNA *Pvt1* identify SMG7 as a key molecular switch for cell survival in response to TNFα.

## 2 Methods

### 2.1 Cell lines and culture conditions

MCF‐7 (RRID: CVCL_0031), NIH 3T3 (RRID: CVCL_0594), and 293T (RRID: CVCL_0063) cells were acquired from ATCC (Manassas, VA, USA). MCF‐7, NIH 3T3, 293T, and immortalized mouse fibroblasts (MF) cells were cultured in DMEM (Gibco, Grand Island, NY, USA) supplemented with 10% FBS superior (Biochrom, Berlin, Germany), 100 U·mL^−1^ penicillin, and 100 μg·mL^−1^ streptomycin (Thermo Fisher Scientific, Waltham, MA, USA) at 37 °C in a humidified atmosphere with 5% CO_2_. Morphology of all cell lines was continuously checked for conformity with ATCC's specifications, and cells were regularly tested for mycoplasma.

### 2.2 Cell viability assays

Unless stated otherwise, 3 × 10^3^ cells were counted by a ViCell cell counter (Beckman Coulter, Brea, CA, USA), seeded in 96‐well plates, and treated with the respective compounds as indicated. For dose–response curves, serial dilutions of respective compounds were prepared in 100 µL medium and cells were added on top in 100 µL medium. Cell viability was assessed by the addition of Resazurin (Sigma, St. Louis, MO, USA) to final concentration of 50 µm, and fluorescence was measured 6 h later at 540 nm excitation/590 nm emission in a PerkinElmer Envision 2104 (PerkinElmer, Waltham, MA, USA) Multilabel plate reader. At least three wells per condition were averaged, and viability is presented as percentage relative to respective control. For growth analysis, 3 × 10^4^ cells were seeded in 12‐well plates and counted by a ViCell cell counter (Beckman Coulter, Brea, CA, USA).

### 2.3 Generation of cell lines

Knockout of *Smg7* in MF was previously described [15]. Overexpression and knockdown cell lines were generated using CRISPRa [19] and CRISPRi [20], respectively. Identification of transcriptional start sites was accomplished using FANTOM5 [21], and top guides (−200) were selected from the UCSC genome browser [22]. Cell pools overexpressing *Adapt33* or *Pvt1* were generated using the CRISPRa guides (Table S1, see Section 2.4. for details). For *Cyld* overexpression cells, the full‐length murine *Cyld* was amplified from parental MF cell cDNA (Table S1) and cloned into the pIREShyg3 (Clontech, Mountain View, CA, USA) construct. After linearizing with SpeI, *Cyld* containing or empty control construct was transfected into MF *Smg7^−/−^* cells. Cell pools were selected with 250 µg·mL^−1^ Hygromycin B (Sigma), and CYLD protein overexpression was confirmed by western blot.

### 2.4 CRISPR activation screen

Thirty‐one upregulated genes in the MF *Smg7^−/−^* cells were selected from our RNAseq data. For each gene, three guides were designed (*Sulf* has 2 TSSs, Table S1). In total, 96 guides were cloned into lenti sgRNA(MS2)_zeo backbone (gift from F. Zhang, Addgene #61427, Watertown, MA, USA) and a mini lentiviral library was used to infect MF‐dCas9‐MS2 screening cells as described [19]. Two days after infection, cells were selected with 200 µg·mL^−1^ Zeocin (Life Technologies, Carlsbad, CA, USA) in a 10 cm dish for 5 days. Then, 3 × 10^5^ cells were treated with 20 ng·mL^−1^ TNFα (Thermo Fisher Scientific) in a 6‐well plate for another 5 days with the replacement of fresh TNFα containing medium every 2 days. Following this, genomic DNA was isolated by phenol‐chloroform extraction from TNFα‐selected and untreated control pools. Triplicate guide sequences were amplified (Table S1) and sequenced on an IonTorrent platform, and deconvolution was performed as described [15].

### 2.5 siRNA knockdown

Mission esiRNAs targeting murine *Smg7* (EMU150861), murine *Pvt1* (EMU193181), murine *Cyld* (EMU031111), human *SMG7* (EHU007301), and *EGFP* (EHUEGFP) were purchased from Sigma. Briefly, 5 × 10^4^ MF or NIH 3T3 cells or 1 × 10^5^ MCF‐7 cells in 500 µL medium were preseeded in 24‐well plates 1 day before. About 30 pmol siRNAs were mixed with 2 µL Lipofectamine RNAiMAX Transfection Reagent (Thermo Fisher Scientific) in 100 µL serum‐free medium, incubated at room temperature for 10 min, and directly added onto the cells. Seventy‐two hours after the transfection, cells were harvested or seeded for subsequent experiments.

### 2.6 qPCR

Total RNA was isolated using InviTrap Spin Cell RNA Mini Kit (Stratec, Birkenfeld, Germany), and 500 ng RNA was used for first‐strand cDNA synthesis via random hexamer primer and AMV Reverse Transcriptase (NEB, Ipswich, MA, USA) following the manufacturer's instructions. The qPCR was carried out on a LightCycler 480 (Roche, Basel, Switzerland) using Power SYBR Green PCR Master Mix (Thermo Fisher Scientific). The program included an initial denaturation step of 95 °C for 10 min and 40 cycles of denaturation at 95 °C for 15 s and combined annealing and extension at 60 °C for 1 min. A melting curve analysis confirmed desired single PCR amplicons. Relative expression levels compared to control conditions were calculated by the comparative 2^−ΔΔCp^ method using *Gapdh* or *Actin* as a reference gene. qPCR primers are listed in Table S1.

### 2.7 Western blotting

Cells were lysed in lysis buffer (63 mm Tris‐HCl, pH 6.8, 10% glycerol, 2% SDS, 2.5% DTT, and 1× protease inhibitor cocktail; Sigma) for 30 min on ice. DNA was shredded with a sonicator and was pelleted for 20 min at max speed centrifugation at 4 °C. The supernatant was mixed with 4× Roti‐Load (Roth) and run on a 6–18% gradient SDS/PAGE gel and transferred onto PVDF membranes (Roth, Karlsruhe, Germany) using electrophoretic semi‐dry western blot transfer system (Phase, Lübeck, Germany). Membranes were blocked with 5% skim milk (Sigma) in TBS‐T for 1 h at room temperature and then incubated with specific primary antibody (dilution 1 : 1000 in 2.5% BSA in TBS‐T or 5% milk in TBS‐T) overnight at 4 °C. Membranes were washed three times for 5 min in TBS‐T before the addition of HRP‐coupled secondary antibody (1 : 2000 in 5% milk in TBS‐T) for 1 h at room temperature. Chemiluminescence detection was conducted using ECL prime western blotting detection reagent (GE Healthcare, Chicago, Illinois, USA) according to the manufacturer's instructions. Experiments were repeated independently at least two times, and antibody specificity was confirmed by comparing bands to molecular weight of the respective target protein. Antibodies used in this paper are listed in Table S1.

### 2.8 Lentiviral transduction

Third‐generation lentiviruses were made using pHCMV‐EcoEnv (Addgene #15802), pRSV‐Rev (Addgene #12253), and pMDLg/pRRE (Addgene #12251) and the respective transfer vectors. Fresh 293T cells were seeded 1 day before with an appropriate number to reach 70% confluency. Plasmids were mixed with X‐tremeGENE HP (Roche) at a ratio of 1 : 3 (DNA : reagent) in serum‐free medium. The transfection complex was incubated at room temperature for 15 min and added dropwise onto 293T cells. Virus supernatant was collected after 72 h, filtered through a 0.45 µm Millex Syringe Filter (Merck Millipore, Darmstadt, Germany), and added to recipient cells. After infection for 48 h, cells were selected under the respective antibiotic to generate pools.

### 2.9 Luminescence assay

Caspase‐8 and caspase‐3/7 activity assays were performed using Caspase‐Glo 8 and 3/7 Assay Systems (Promega, Madison, WI, USA) following the manufacturer's instructions. Briefly, 1.5 × 10^4^ *Smg7^−/−^* or parental MF cells per well were seeded 1 day before in a 96‐well plate. On the second day, cells were exposed to 20 ng·mL^−1^ TNFα (Thermo Fisher Scientific) for 8 h or left untreated. The medium was removed, and cells were washed twice with PBS. Fifty microlitre reagent and 50 µL PBS were added and incubated at room temperature with continuous shaking. After 20 min, luminescence was recorded on an Envision 2104 Multilabel plate reader (PerkinElmer). Empty wells served as blanks and were subtracted from all values.

### 2.10 3D spheroid culture

Five hundred parental MF cells and 1000 *Smg7^−/−^* cells per well were seeded into Corning 4515 plates (Corning, NY, USA), grown for 4 days, and treated with 20 ng·mL^−1^ TNFα and 10 µm SC‐514 for 2 days, with replacement of medium after 1 day. Spheroids were stained with 1 µg·mL^−1^ propidium iodide (PI) for 1 h and imaged in a PerkinElmer Operetta High Content Imaging System using brightfield (BF) and 535/30 nm excitation and 595/70 nm emission channel.

### 2.11 Quantitative mass spectrometry

1 × 10^7^ *Smg7^−/−^* and parental MF cells per replicate (*n* = 3) were lysed in 8 m urea in 0.1 m Tris/HCl pH 8.5 using a Precellys homogenizator (Bertin Technologies, Montigny‐le‐Bretonneux, France). Equal amounts were proteolysed using a modified FASP procedure [23]. Briefly, after reduction and alkylation using DTT and IAA, the proteins were centrifuged on Microcon centrifugal filters (Vivacon 500 30 kDa, Sartorius, Goettingen, Germany) and washed 3× with 8 m urea in 0.1 m Tris/HCl pH 8.5 and twice with 50 mm ammonium bicarbonate. The proteins were digested for 2 h at room temperature using 0.5 µg Lys‐C (Wako Chemicals, Neuss, Germany) and for 16 h at 37 °C using 1 µg trypsin (Promega). Peptides were eluted by centrifugation (10 min at 14 000 ***g***), acidified with 0.5% TFA, and stored at −20 °C. Approximately 0.5 µg of peptides per sample was measured on a Q‐Exactive HF mass spectrometer online coupled to an Ultimate 3000 nano‐RSLC (Thermo Scientific, Waltham, MA, USA) in data‐independent acquisition (DIA) mode as described previously [24]. The recorded raw files were analyzed using the spectronaut pulsar software [25] with a peptide and protein identification false discovery rate setting of < 1%, using an in‐house mouse spectral meta library which was generated using proteome discoverer 2.1 (Thermo Scientific), the Byonic search engine (Protein Metrics, Cupertino, CA, USA), and the SwissProt Mouse database (release 2016_02). Quantification was based on MS2 area levels of all unique peptides per protein fulfilling the percentile 0.3 setting. Normalized protein quantifications were exported and used for calculations of fold changes and significance values.

### 2.12 RNA sequencing and analysis

Independent replicates of *Smg7^−/−^* and parental controls (*n* = 3) were harvested, and RNA extracted using the RNeasy Mini Kit (Qiagen, Hilden, Germany) and ribosomal RNA (rRNA) subtractive sequencing was performed as 150 bp paired‐end runs on Illumina HiSeq 4000 platform. RNAseq analysis was performed on Galaxy with alignment against mouse genome version mm10.gtf using Salmon transcript quantification [26]. Gencode vM17 transcripts were used as reference transcriptome with transcript‐to‐gene mapping. Sequence data were visualized with Broad IGV. Biotype assignments were retrieved from Ensembl/Gencode through the Biomart function. Transcript counts below 1 were excluded from further analysis. Significance between *Smg7^−/−^* and parental cells for each biotype was determined using deseq2 [27]. Differential gene expression analysis was performed on rRNA‐depleted samples with tophat and cufflinks [28] using default settings on Galaxy and mouse genome version mm10. tophat alignment to the file gencode.vM12.lncRNA_transcripts.fa was performed to determine percent transcript mapping to lncRNA. Cuffdiff was used to calculate FPKM levels for each gene with Cuffdiff gene level determinations with the default settings.

### 2.13 Gene set enrichment analysis

Gene set enrichment analysis (GSEA) was performed using top upregulated and downregulated (*n* = 100 each) genes as described [29].

### 2.14 SMG7 and CYLD correlation analysis

Cumulative CCLE [30] data (Expression Public 19Q1) were downloaded from depmap [31]. Linear regression and Pearson coefficient analyses were performed in graphpad prism (GraphPad Software, San Diego, CA, USA).

### 2.15 Association between SMG7 and CYLD and patient survival

Kidney renal clear cell carcinoma (KIRC) transcriptome data and clinical information were downloaded via The Cancer Genome Atlas [32] on December 15, 2019. The correlations between gene expression levels were calculated by Pearson's test in r version 3.53 (https://www.r‐project.org/). A Kaplan–Meier plot was applied to identify the association between SMG7 expression and patient survival. Log‐rank test and univariate Cox regression were used to determine significance. Data were visualized using ggplot2 [33]. Briefly, all tumor samples were centered into nearest neighbor values by their expression level of *CYLD*. Each dot in the plot represents averaged SMG7 expression level of 40 unique tumor samples.

### 2.16 Statistics/data analysis

General data visualization and statistics were performed in graphpad prism. If not stated otherwise, Student's two‐tailed, unpaired *t*‐test against respective control conditions was used to determine significance.

## 3 Results

### 3.1 Apoptosis‐resistant *Smg7^−/−^* cells upregulate noncoding RNAs

**Fig. 1:**
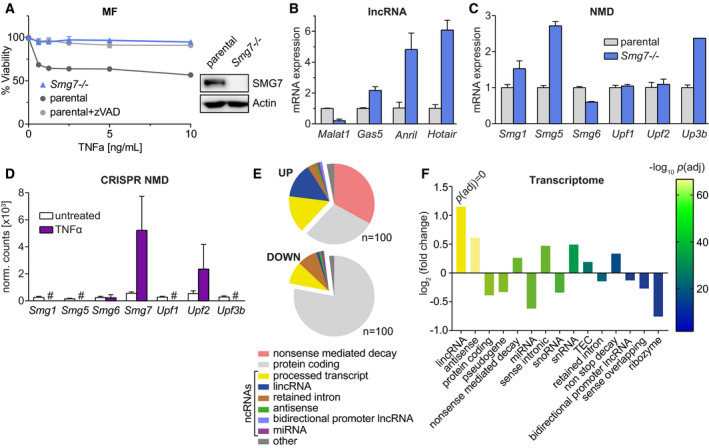
*Smg7* ablation in MF cells drives TNFα resistance and upregulation of NMD and noncoding RNAs. (A) TNFα dose–response curves in *Smg7^−/−^* cells compared to parental MF cells (parental) with caspase inhibitor Z‐VAD‐FMK 10 µm (zVAD) and validation of ~127 kD protein absence by western blot. Viability is represented as mean ± SEM of *n* = 4 technical replicates. The experiment was repeated independently *N* = 3 times with similar results and a representative example is shown. (B, C) Gene expression analysis by qPCR of hallmark lncRNA targets and a panel of NMD regulators in *Smg7^−/−^* relative to parental MF cells. Expression data are shown as mean ± SD of *n* = 3 technical replicates. (D) Normalized read counts of NMD regulators from a pan‐genomic CRISPR mutagenesis screen in MF cells after TNFα selection. Data represent mean ± SEM of *n* = 2–5 guides per gene according to Ref. [15]. (E, F) Differential gene expression of *Smg7^−/−^* compared to parental MF cells. RNA of *n* = 3 technical replicates were deep sequenced, and Gencode transcripts and significance were evaluated using deseq2. (E) Biotype classification of the top 100 up‐ and downregulated genes. (F) Global analysis of differentially expressed biotypes of *Smg7^−/−^* transcripts compared to parental, evaluated by log_2_ fold change and significance. #, not detected, ncRNAs, noncoding RNAs, *P*(adj), *P*‐value adjusted for multiple hypothesis testing. See text for additional abbreviations.

Depending on the cellular context, TNFα can either induce cell death or activate the NF‐κB pathway. We have shown that *Smg7* ablation in immortalized mouse fibroblasts (MF) protects against TNFα‐induced apoptosis but not chemically induced cell death [15]. We re‐analyzed *Smg7^−/−^* MF cells for TNFα sensitivity and observed Z‐VAD‐FMK (zVAD) rescue, demonstrating caspase involvement (Fig. 1A). Independent knockdown of *Smg7* by CRISPR interference [20] in NIH 3T3 cells (*Smg7*KD) recapitulated the TNFα‐resistant phenotype (Fig. S1A). In both instances, a consistent fraction of cells was insensitive to TNFα‐induced apoptosis, likely due to impeded signaling in cellular aggregates.

Next, we tested whether *Smg7* loss affects TNFα signaling exclusively or interferes with other forms of cell death. We treated MF cells with TNF‐related apoptosis‐inducing ligand (TRAIL), TNF‐related weak inducer of apoptosis (TWEAK), and lipopolysaccharide (LPS), but observed insensitivity to cell death in parental and *Smg7^−/−^* cells, irrespective of cycloheximide or IFNγ sensitization (Fig. S1B). A panel of chemotherapeutics showed minor protection in *Smg7^−/−^* cells against doxorubicin and staurosporine and partial zVAD‐independent sensitivity to paclitaxel (Fig. S1C). This shows increased viability due to *Smg7* mutation is largely stimulus‐dependent and primarily affects TNFα signaling.

Classical targets of NMD include PTC‐containing transcripts and lncRNAs. We analyzed hallmark lncRNAs for downstream effects due to the loss of *Smg7* by quantitative PCR (qPCR). Three transcripts, *Gas5*, *Anril*, and *Hotair,* were elevated ~2‐ to 6‐fold, while *Malat1* was decreased (Fig. 1B). We suspected that these relatively minor changes were due to functional redundancy in the NMD complex or as compensation for SMG7. However, analysis of a panel of NMD effectors showed only minor increases in *Smg5* and *Upf3b* transcripts, suggesting negligible compensation (Fig. 1C). We therefore examined whether ablation of these same NMD factors could also protect against TNFα using data from our pan‐genomic mutagenesis screen in the same cells [15]. Only basal guides directed against *Smg7* and *Upf2* were found to be enriched. This suggests that resistance to TNFα‐induced apoptosis is mediated by specialized function of SMG7 rather than by global disruption of NMD.

We therefore investigated whether particular classes of transcripts are affected in*Smg7^−/−^* cells using RNA sequencing. We grouped resulting transcripts by biotype and observed that prototypical NMD targets are most enriched in the top 100 upregulated transcripts. Notably, protein‐coding genes, miRNAs, and processed transcripts (consisting of lncRNAs and antisense RNAs) comprise 45% of the top upregulated transcripts (Fig. 1E). The majority (78%) of downregulated transcripts, in contrast, are almost exclusively protein‐coding genes.

Strikingly, global differential expression analysis revealed long intergenic noncoding RNAs [lincRNA; Fig. 1F; *P*(adj) ≈ 0.0000] and antisense transcripts [*P*(adj) = 1.84E‐67] to be most significantly upregulated, while protein‐coding genes showed only a nominal decrease (Fig. 1F). Separate subclasses of lncRNAs, including small nuclear RNAs (snRNA), sense intronic, and nonstop decay transcripts, were similarly elevated. Global NMD targets, in contrast, only showed a limited increase. As expected, the miRNA class of transcripts was decreased [*P*(adj) = 5.65E‐22], consistent with a lncRNA role as molecular sponges [34]. Gene set enrichment analysis confirmed cancer pathway features and downregulation of apoptosis genes (Fig. S2). A separate proteomic analysis of *Smg7^−/−^* cells also showed accumulation of cytokine response factors (such as Mgst3) and caspase‐1 (Fig. S3). Proteins that negatively regulate viruses also featured, possibly as a consequence of expressing unconventional transcripts.

Pseudogenes mRNAs contain PTCs that target them for NMD; therefore, these would be expected to be elevated [35]. However, pseudogene transcripts were largely reduced in *Smg7^−/−^* cells (Fig. 1F). Together, these data suggest that SMG7 preferentially targets processed transcripts such as lncRNAs over PTC‐containing RNAs. Additionally, as discussed in Section 3.4., potent regulation of individual transcripts argues for selective target RNA regulation by SMG7.

### 3.2 *Smg7^−/−^* cells show decreased caspase activity and CYLD levels

**Fig. 2:**
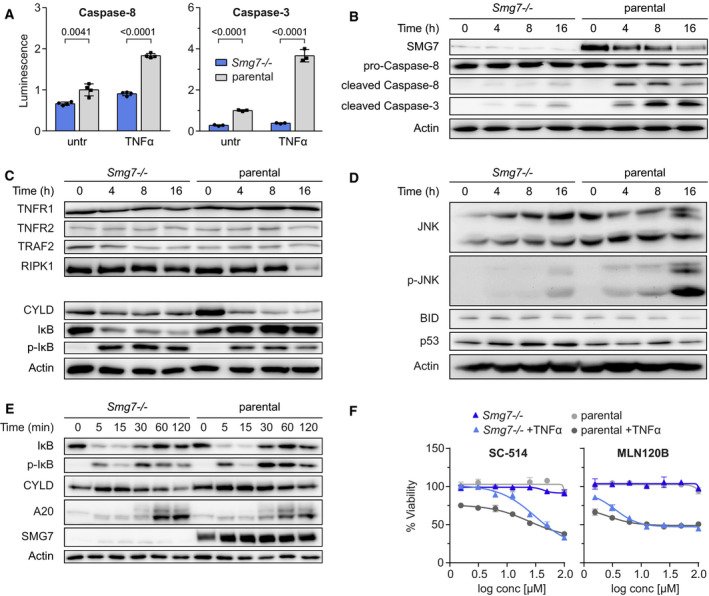
Reduced caspase activity and CYLD expression in *Smg7^−/−^* cells. (A) Caspase‐8 and caspase‐3 activity in *Smg7^−/−^* compared to parental MF cells in untreated (untr) conditions and after 20 ng·mL^−1^ TNFα treatment for 8 h. Luminescence intensity in arbitrary units is shown as mean ± SD of *n* = 3 or 4 technical replicates. The experiment was repeated independently *N* = 2 times with similar outcomes. (B–E) TNFα pathway related expression levels determined by western blot in *Smg7^−/−^* and parental MF cells after 20 ng·mL^−1^ TNFα treatment at indicated time points. Western blots were divided into four groups: (B) pro‐ and cleaved caspase‐8 and caspase‐3 proteins, (C) TNF receptor and NF‐κB‐related proteins, (D) mitochondrial apoptosis‐associated proteins, and (E) short interval TNFα treatment of NF‐κB proteins. (F) Dose–response curves of IKK inhibitors SC‐514 or MLN120B in *Smg7^−/−^* compared to parental MF cells. Cells were pretreated with IKK inhibitors for 2 h followed by 20 ng·mL^−1^ TNFα addition for 36 h. Viability is represented as mean ± SEM of *n* = 3 technical replicates.

Extrinsic apoptosis is executed by caspases in the death‐inducing signaling complex (DISC). To determine where in the TNFα signaling cascade SMG7 acts, we first examined caspase‐8 and ‐3 activity. Using a luminescence assay, we observed that caspase‐8, and more significantly, caspase‐3 activity was strongly decreased in *Smg7^−/−^* cells stimulated with TNFα (Fig. 2A). Diminished activity was corroborated in a TNFα time course by western blot showing a reduction of the pro‐caspase form and accumulation of the mature cleaved enzyme in parental cells. These were largely absent in*Smg7^−/−^* cells (Fig. 2B).

In light of reduced caspase activity, we inferred that SMG7 must functionally act on or upstream of these enzymes. We examined TNFα signaling receptors TNFR1 and ‐2, but detected no changes in protein expression to account for apoptosis inhibition (Fig. 2C). Receptor‐associated complex I factors TRAF2 and RIPK1 expression were similarly unaffected; however, CYLD deubiquitinase showed a striking decrease in basal levels in *Smg7^−/−^* cells compared to parental cells (Fig. 2C). CYLD acts at the interface between cell death and survival pathways. Therefore, we tested and confirmed a strong increase in phosphorylated‐IκB and its associated degradation, indicating NF‐κB pathway activation, in *Smg7^−/−^* cells.

Activation of Jun‐kinase (phospho‐JNK) in parental cells [36] and other effectors of the mitochondrial cell death pathway P53 and BID remained unchanged compared to parental cells. Together, these data confirm that SMG7 acts upstream of JNK/caspases but downstream of surface receptors to sensitize cells to TNFα.

CYLD is a tumor suppressor that deubiquitinates K63 chains directly downstream of TNFα receptors. When CYLD is diminished, the NF‐κB survival pathway is activated [37]. We therefore tested NF‐κB activation in a short interval time course and observed that the classical activation‐feedback inhibition–activation response was unaltered in *Smg7^−/−^* cells following TNFα administration (Fig. 2E). This indicates that functional TNFα signaling is enabled through its receptors. Moreover, A20, a transcriptional target and NF‐κB effector, was strongly increased in *Smg7^−/−^* cells. Therefore, we tested whether TNFα‐resistance in *Smg7^−/−^* cells is dependent on NF‐κB using pharmacological inhibitors SC‐514 and MLN120B. Cell viability was strongly decreased upon treatment in the presence of TNFα, with *Smg7^−/−^* cells requiring higher concentrations of both inhibitors to achieve equivalent levels of cell death (Fig. 2F). These results demonstrate that resistance to TNFα‐induced apoptosis in *Smg7^−/−^* cells is mediated by CYLD/NF‐κB.

### 3.3 CYLD and SMG7 coordinate apoptosis sensitivity

**Fig. 3:**
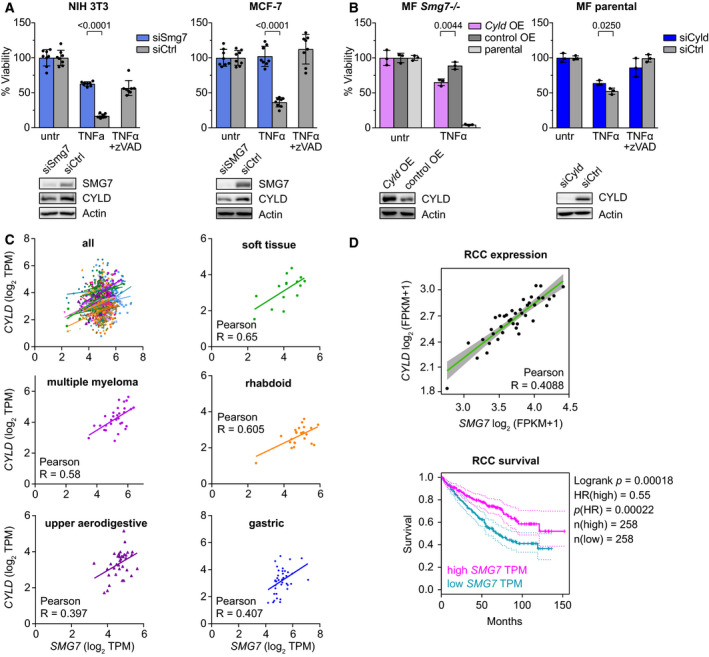
Coordinated CYLD and SMG7 expression levels determine apoptosis sensitivity. (A) Viability of siRNA‐mediated *Smg7* knockdown (siSmg7) compared to control siRNA (siCtrl) in NIH 3T3 (left) and human MCF‐7 cells (right) exposed to 10 ng·mL^−1^ TNFα with 10 µm Z‐VAD‐FMK (zVAD) control. Western blots of SMG7 knockdown efficiency and CYLD expression. (B) Viability of *Cyld* overexpression (*Cyld* OE) compared to empty vector control (control OE) in *Smg7^−/−^* MF cells or parental MF cells treated with 5 ng·mL^−1^ TNFα (left). Viability of siRNA *Cyld* knockdown (siCyld) compared to control (siCtrl) in parental MF cells exposed to 10 ng·mL^−1^ TNFα with 10 µm zVAD control (right). Western blots of CYLD overexpression and knockdown efficiency. (C) Relationship between *CYLD* and *SMG7* expression levels in 1164 human cancer cell lines in the CCLE database determined by linear regression. Selected cell lines with indicated tissue of origin and high degree of association by Pearson's *R*‐value are shown. (D) Correlation of *CYLD* and *SMG7* expression in renal cell carcinoma (RCC, upper panel) and Kaplan–Meier plot of *SMG7* expression and survival in RCC patients (lower panel). Viability data are represented as mean ± SD of *n* = 8 (A) or 3 (B) technical replicates of at least *N* = 2 (A) independent repetitions with similar outcomes. untr, untreated; TPM, transcripts per million; FPKM, fragments per kilobase of transcript per million; HR, hazard ratio.

We hypothesized that CYLD levels are qualified by SMG7 and evaluated this relationship in other cell types. Forced knockdown of SMG7 by siRNA (siSmg7) in NIH 3T3 and MCF‐7 breast cancer cells restored viability upon TNFα treatment (Fig. 3A). In a striking similarity to MF cells, SMG7 knockdown in both cell lines resulted in CYLD downregulation and TNFα resistance, suggesting conservation of this relationship. We therefore tested whether ectopic *Cyld* overexpression (*Cyld* OE) in *Smg7^−/−^* cells could override TNFα resistance and observed a partial resensitization compared to empty vector control (Fig. 3B). Correspondingly, *Cyld* siRNA knockdown (siCyld) partially increased resistance of parental MF cells to TNFα (Fig. 3B). Together, these results indicate the *SMG7*/*CYLD* relationship is conserved and that direct manipulation of CYLD expression influences apoptosis sensitivity.

We chose to examine whether this relationship extended further. We therefore compared *CYLD* and *SMG7* expression levels in 1164 human cancer cell lines [31] and found a comprehensive positive correlation as well as marked Pearson correlations for soft tissue (*R* = 0.65; Fig. 3C), multiple myeloma (*R*= 0.58), rhabdoid (*R* = 0.605), upper aerodigestive (*R* = 0.397), and gastric cancers (*R* = 0.407). A moderate correlation (*R* = 0.41) was observed in primary renal cell carcinoma samples [32]; however, RCC cells have established susceptibility to TNF family‐induced apoptosis through the TNF receptors [38, 39] and plasma TNFα levels are a prognostic indicator for RCC. Higher TNFα levels may therefore indicate refractory, or NF‐κB activating, tumors. Consistent with this, Kaplan–Meier analysis of 516 KIRC‐TCGA patients revealed a strong correlation between elevated SMG7 expression and survival [*P*(HR) = 0.00022; Fig. 3D].

### 3.4 SMG7 regulates anti‐apoptotic lncRNAs *Pvt1* and *Adapt33*

TNFα sensitivity in *Smg7^−/−^* cells is incompletely rescued by *Cyld* overexpression (Fig. 3B). To determine whether RNA regulation plays a role in this process, we analyzed ribosomal RNA‐depleted transcripts from *Smg7^−/−^* and parental MF cells. Other NMD factors are mostly unchanged in mutant cells (Fig. 1C); thus, we expected RNAs specifically degraded by SMG7 to be upregulated. Surprisingly, of the top 332 significant known genes, only 38 were found to be upregulated compared to controls (Fig. S4A). This gene set is enriched with small nucleolar RNA host genes (lncRNAs *Snhg1, ‐5, ‐6, ‐12, ‐15*), known oncogenes (*Pvt1*, *Klf4*), and known apoptosis resistance genes (*Nupr1*, *Sulf1*, *Tnfrsf11b*). Alignment to lncRNA transcripts (Gencode vM12) showed that 13.5% of transcripts from *Smg7^−/−^* cell reads mapped to lncRNA transcripts, while only 7.3% of transcripts from parental cells were a match (Fig. S4B).

**Fig. 4:**
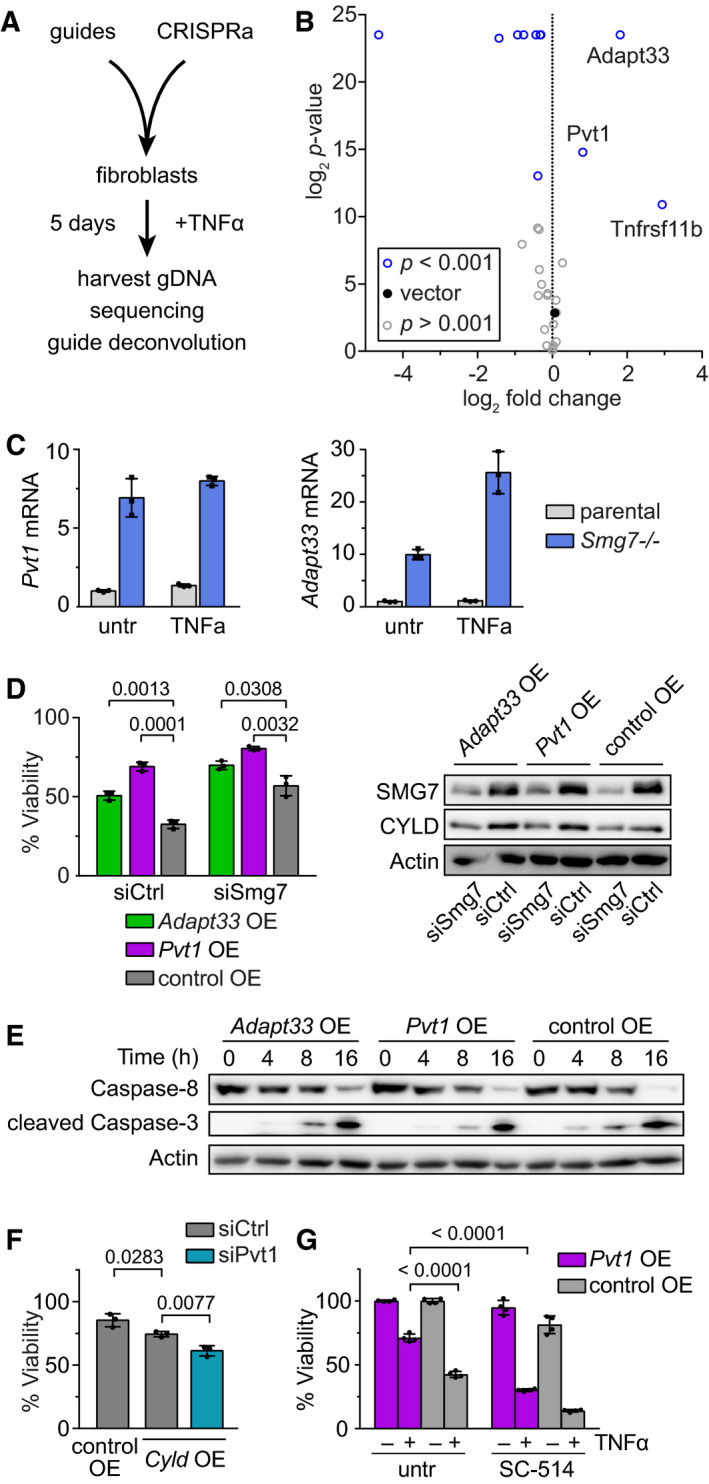
SMG7 regulates anti‐apoptotic lncRNAs *Pvt1* and *Adapt33*. (A) CRISPR activation screen workflow. A mini‐library was designed for upregulated genes from *Smg7^−/−^* MF cells. Parental cells expressing CRISPRa components were infected with the pooled guide library followed by 20 ng·mL^−1^ TNFα treatment for 5 days. Genomic DNA was extracted from surviving cells and guide sequences were amplified for sequencing and deconvolution. (B) Identification of anti‐apoptotic genes from *Smg7^−/−^* cells. Guides from positively scoring genes are displayed in the upper right corner. (C) qPCR analysis of *Pvt1* and *Adapt33* expression in parental and *Smg7^−/−^* MF cells stimulated with 20 ng·mL^−1^ TNFα for 8 h or untreated (untr). Expression data are shown as mean ± SD of *n* = 3 technical replicates. (D) Viability of *Pvt1* or *Adapt33* overexpression cells (*Pvt1* OE, *Adapt33* OE) compared to empty OE vector control (control OE) cells upon 5 ng·mL^−1^ TNFα treatment for 36 h. Additional siRNA *Smg7* knockdown (siSmg7) compared to control siRNA (siCtrl). Viability is shown as mean ± SD of *n* = 3 technical replicates. A typical result of *N* = 2 independent repetitions is shown. Western blot of *Pvt1* OE, *Adapt33* OE, and control OE cells with siSmg7 knockdown and corresponding CYLD protein levels. (E) Western blot corresponding to (D) showing caspase‐8 and cleaved caspase‐3 protein levels following 20 ng·mL^−1^ TNFα stimulation at respective time points in *Pvt1* OE, *Adapt33* OE, and control OE cells. (F) Viability of siRNA knockdown of *Pvt1* (siPvt1) compared to control (siCtrl) in *Cyld* OE and empty OE vector control (control OE) cells treated with 10 ng·mL^−1^ TNFα. Viability is shown as mean ± SD of *n* = 3 technical replicates. A representative result of *N* = 2 independent repetitions is shown. (G) Viability of *Pvt1* OE compared to empty OE vector control cells (control OE) treated with 10 µm SC‐514 in presence or absence of TNFα for 48 h. Viability data represent mean ± SD of *n* = 4 technical replicates. untr, untreated.

Many lincRNAs have not yet been functionally characterized. We wondered whether top enriched genes, including protein‐coding genes, could functionally recapitulate *Smg7^−/−^* resistance to TNFα. A CRISPR activation [19] library was designed targeting the promoters of top upregulated genes (Fig. 4A and Fig. S4A). We selected only genes with a clear transcription start site (TSS) and used a tiling function within −200 bp of the TSS. Parental MF cells were transduced with the pooled CRISPRa mini‐library and treated with TNFα for 5 days to induce extrinsic apoptosis. Genomic DNA from surviving cells was harvested for deep sequencing, and guides were deconvoluted utilizing encore software [15]. After multiple hypothesis correction, only two lncRNAs were found to significantly protect against TNFα challenge: the oncogene plasmacytoma variant translocation 1 (*Pvt1*; *P* = 0.000865, FDR = 0.0031) and *5430416N02Rik*, also known as *Adapt33* (*P *< 0.0001, FDR < 0.0001; Fig. 4B). The decoy TNF receptor *Tnfrsf11b* also displayed protection, likely via interference at TNFRSF1A/B signaling receptors. However, given downstream NF‐κB activation in *Smg7^−/−^* cells (Fig. 2C,E), we surmised that TNF receptor signaling at the membrane is not substantially impeded. Thus, we focused our investigation on the two lncRNAs.

*Adapt33*is a stress‐induced lncRNA induced by hydrogen peroxide or staurosporine [18]. We examined lncRNA expression by qPCR in *Smg7^−/−^* cells and observed a 7‐ and 10‐fold increase in *Pvt1* and *Adapt33* transcripts, respectively, that increased upon TNFα treatment (Fig. 4C). We therefore generated *Pvt1* and *Adapt33* overexpression cell lines (OE; Fig. S4C) and evaluated resistance to TNFα. Compared to control cells containing an empty vector, *Pvt1* OE cells were significantly more viable at 5 ng·mL^−1^ TNFα (*P* = 0.0001; Fig. 4D). *Adapt33* OE also displayed protection, albeit at lower levels (*P *= 0.0013). We reasoned that incomplete protection was due to persistent SMG7 surveillance of overexpressed transcripts. Inclusion of siRNA directed against *Smg7* (siSmg7; Fig. S4D) substantially potentiated *Pvt1* OE and *Adapt33* OE resistance to TNFα (Fig. 4D). However, we determined that *Pvt1* OE and *Adapt33* OE, while individually protecting against TNFα, do not depress CYLD levels detectably (Fig. 4D). Caspase‐8 and ‐3 cleavage in both cell lines was comparable to parental cells (Fig. 4E). However, a synergistic sensitization to TNFα was observed upon *Pvt1* knockdown in *Cyld* overexpression cells (Fig. 4F). Thus, protection afforded by these lncRNAs likely mechanistically complements the SMG7‐CYLD relationship. Nevertheless, the NF‐κB inhibitor SC‐514 sensitized *Pvt1* OE cells to TNFα (Fig. 4G), suggesting that *Pvt1* OE resistance also relies on the activation of NF‐κB pathway.

### 3.5 Pharmacological sensitization of *Smg7^−/−^* cells to TNFα in 3D spheroid model

**Fig. 5:**
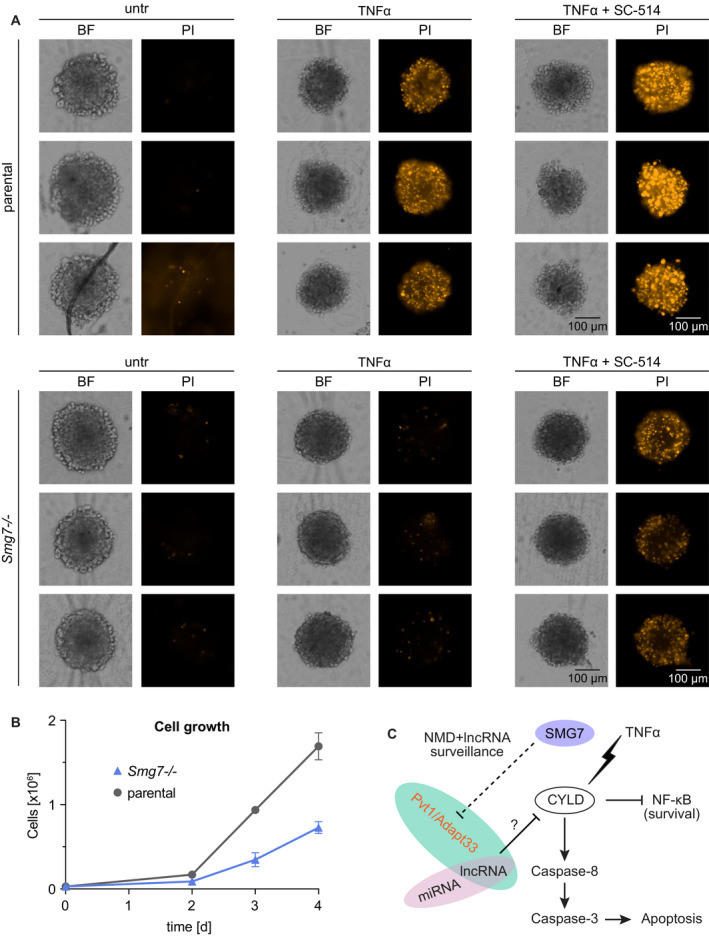
Growth and pharmacological sensitization of *Smg7^−/−^* cells to TNFα in 3D spheroid model. (A) Three‐dimensional spheroids of *Smg7^−/−^* and parental MF cells were grown for 4 days and treated with 20 ng·mL^−1^ TNFα and addition of 10 µm SC‐514 for 48 h. Propidium iodide (PI) staining indicates dead cells next to corresponding bright field images (BF). Scale bar = 100 µm. untr, untreated. (B) Growth characteristics of *Smg7^−/−^* compared to parental MF cells in culture. Counted cells are shown as mean ± SD of *n* = 3 technical replicates and a typical result of *N* = 3 independent repetitions is shown. (C) Modeling SMG7 effect on oncogenic networks. Following TNFα treatment, loss of SMG7 surveillance of NMD and lncRNA targets leads to inhibition of CYLD by an uncharacterized mechanism and subsequent activation of the NF‐κB survival pathway. By contrast, unmodified cells with normal SMG7/CYLD have reduced *Pvt1* and *Adapt33* levels and undergo apoptosis upon TNFα treatment.

TNFα has pluripotent effects on tumorigenesis and cancer progression and an autocrine function in the tumor microenvironment. To mimic *in vivo* tissue and cellular communication, we examined the effect of *Smg7* deletion and TNFα in a 3D spheroid model [40] (Fig. 5A). We observed that *Smg7^−/−^* cells have a growth disadvantage compared to parental cells (Fig. 5B). Seeding 500 parental and 1000 *Smg7^−/−^* cells compensated for this difference yielding comparable spheroids. TNFα sensitivity of parental cells indicated by propidium iodide (PI) was preserved in this environment, while synthetic lethality with 10 µm SC‐514 IKK inhibitor was increased (Fig. 5A). TNFα treatment of *Smg7^−/−^* cells resulted in compacted, sharply defined spheroids, but did not affect viability. Pharmacological sensitization of *Smg7^−/−^* cells with SC‐514 nevertheless produced comparable cell death to TNFα treatment of parental cells.

## 4 Discussion

TNFα plays dynamic but often paradoxical roles in human malignancies [41]. Systemic TNFα can activate inflammation and pro‐survival pathways via NF‐κB, while higher local concentrations, that is, monocyte presentation or natural killer induced cytotoxicity, can induce apoptosis in tumors [42, 43]. Fascinatingly, these contradictory outcomes are both triggered via by TNFα binding to its cognate receptors TNFRSF1/2 but ultimately decided by cellular factors directly downstream. The abundance of these factors in localized complexes is crucial in this decision. In particular, TNFα cytotoxicity is strongly influenced by the NF‐κB pathway and associated factors [44].

In this study, we determined that SMG7 dictates levels of CYLD deubiquitinase and that loss of SMG7 leads to lower CYLD levels and increased cell survival upon TNFα challenge. The primary consequence of CYLD downregulation is the activation of the NF‐κB pathway. This implies that cancers associated with constitutive NF‐κB activation are more likely to be susceptible to SMG7 levels. Indeed, NF‐κB therapeutics for prostate cancer, for which SMG7 was found to be a novel risk factor [14], shows promise in modulating tumorigenesis and progression [45]. Paracrine application of TNFα is known to induce apoptosis and cell cycle arrest in prostate cancer cells [46]. A direct relationship was also observed by *CYLD* ablation in testicular cells, resulting in NF‐κB activation and aberrant expression of anti‐apoptotic genes [47]. These results are entirely consistent with the phenotype of *Smg7^−/−^* cells reported here, including resistance to TNFα and pharmacological sensitization to the combination of NF‐κB inhibitors and TNFα in monolayers and 3D spheroids.

While the mechanism of SMG7 activity directly on specific target RNAs remains to be fully established, the present results demonstrate that, in addition to NMD transcripts, SMG7 targets a unique subset of lncRNAs. Whether SMG7 targets these transcripts on its own or in concert with other NMD proteins remains to be determined. SMG7 alone may contain the capacity to degrade RNA transcripts via interaction with CNOT8 [11] and can also degrade 3′ UTR length‐dependent mRNA via UPF1/SMG7‐dependent miRNA‐mediated mRNA decay pathway [48]. Screening results of other NMD factors, in contrast to *Smg7*, did not reveal protection against TNFα (Fig. 1C). Thus, our data support a model of specialized transcript degradation over a generalized contribution to NMD, with individual lncRNAs and mRNAs showing profound differences.

One of the most highly upregulated transcripts in *Smg7^−/−^* cells is *Pvt1*. *Pvt1* overexpression, as well as the lncRNA *Adapt33*, was sufficient to protect MF cells from TNFα‐induced apoptosis. The relationship between SMG7, *Pvt1,* and CYLD is mechanistically complex (Fig. 5C). *PVT1* was originally identified in Burkitt's lymphoma. It is located chromosomally adjacent to and regulates MYC [49], which in turn is responsible for the majority of Burkitt's lymphoma. *SMG7* is highly expressed in cells derived from Burkitt's lymphoma [30]. *PVT1* depletion in HCT 116 colon cancer cells dramatically compromised their ability to form tumors, as high MYC protein levels are dependent on *PVT1* [49]. *PVT1* lncRNA does not directly regulate CYLD, but CYLD is implicated in controlling MYC via JNK [50], providing a hint to this relationship.

*Adapt33* was reported as an oxidant‐inducible and apoptosis‐associated RNA [51]; however, its function has not yet been demonstrated. Here, we report that *Adapt33* mRNA level is upregulated in *Smg7^−/−^* MF cells and overexpression of *Adapt33* by CRISPRa system can give protection to MF cells against TNFα‐induced apoptosis. *Adapt33* mRNA is also increased in MF cells after TNFα treatment in our study. The underlying anti‐apoptotic mechanism of *Adapt33*, however, is still uncharacterized.

Long noncoding RNAs can impact multiple pathways simultaneously. As testament to this complexity, *PVT1*‐encoded lncRNA or derived microRNAs have oncogenic functions [52]. Additionally pointing to miRNAs, *CYLD* transcripts have been shown to be targeted by miR‐362‐5p and miR‐19 [53, 54]. Thus, the possibility exists that other lncRNAs upregulated in *Smg7^−/−^* cells may be processed to miRNAs that target *Cyld*. Indeed, a precedent already exists as miR‐20a induces cisplatin resistance in human gastric cancer cells by targeting *CYLD* [55]. miRNAs from the *PVT1* locus have also been suggested to have a regulatory function [52]. Similarly, HOTAIR overexpression maintains NF‐κB expression and platinum chemoresistance [56], although a link via CYLD has not been explored. Certainly, the role of such multifactorial transcripts in cancer is being further dissected through large‐scale genomic studies [57].

## 5 Conclusions

A major open question in oncology is how lncRNAs influence cell death pathways. Here, we show that loss of the NMD factor SMG7 in several cell types uniquely protects against TNFα‐induced apoptosis. Strikingly, this phenotype is mediated by two different oncogenic factors. *CYLD* is positively correlated with SMG7, while overexpression of *Pvt1* increases cell viability toward TNFα treatment. Taken together, our findings support a novel role of SMG7 in TNFα‐induced apoptosis by regulating *Pvt1* and the tumor suppressor CYLD and suggest a comprehensive role for regulation of NF‐κB by an NMD factor.

## Conflict of interest

The authors declare no conflict of interest.

## Author contributions

JAS and LY designed the project. LY, VANK, and SP conducted experiments. JAS, LY, and VANK analyzed data, produced figures and tables, and wrote the manuscript. XB analyzed patient survival and primary renal cell carcinoma samples. YA assisted with oncogenic models. JM‐P and SMH acquired and analyzed quantitative mass spectrometry data. All authors have read and approved the final manuscript.

## Supporting information


**Fig. S1.** Effects of *Smg7* ablation on cell death inducers.

**Fig. S2.** Gene Set Enrichment Analysis in *Smg7^−/−^* cells.

**Fig. S3.** Quantitative mass spectrometry analysis of *Smg7^−/−^* compared to parental MF cells.

**Fig. S4.** Transcriptional changes in *Smg7^−/−^* cells.

**Table S1.** Primers, guides and antibodies used in this study.

**Table S2.** Quantitative mass spectrometry data.

## Data Availability

RNAseq data from this study have been deposited under the accession number PRJNA610469 (https://dataview.ncbi.nlm.nih.gov/object/PRJNA610469) at the NCBI Sequence Read Archive (SRA).
