# Supplementary material for: Nonsense‐mediated decay factor SMG7 sensitizes cells to TNFα‐induced apoptosis via CYLD tumor suppressor and the noncoding oncogene Pvt1
Source: Mol Oncol. 2020 Jul 13;14(10):2420–35. doi: 10.1002/1878-0261.12754 (PMC7530794; doi:10.1002/1878-0261.12754)
Supplement: Supplementary file 1 — Fig. S1. Effects of Smg7 ablation on cell death inducers. [file MOL2-14-2420-s001.pdf]

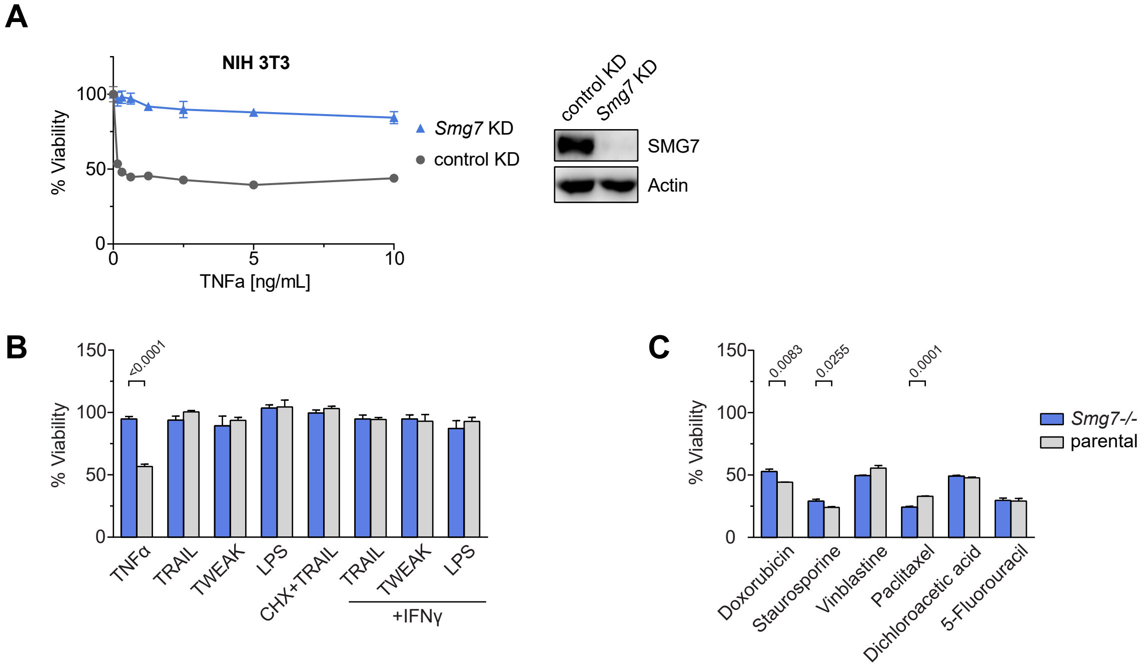

Fig. S1. Effects of *Smg7* ablation on cell death inducers.

(A) TNFα dose-response curves of *Smg7* CRISPRi knockdown (*Smg7* KD) compared to empty KD vector control NIH 3T3 cells (control KD). Viability data represent mean ± SEM of n = 4 technical replicates of two independent experiments.

(B) Viability of *Smg7 -/-* compared to parental MF cells (parental) against apoptosis inducers: 10 ng/mL tumor necrosis factor (TNFα), 100 ng/mL TNF-related apoptosis-inducing ligand (TRAIL), 12.5 ng/mL TNF-like weak inducer of apoptosis (TWEAK), 10 µg/mL lipopolysaccharide (LPS), co-treatment with 20 pg/mL cycloheximide (CHX) or 20 ng/mL interferon gamma (IFNγ).

(C) Viability of *Smg7 -/-* compared to parental MF cells (parental) against chemotherapeutic drugs: 20 µM Doxorubicin, 2 µM Staurosporine, 0.05 µM Vinblastine, 0.4 µM Paclitaxel, 250 mM Dichloroacetic acid, 50 µM 5-Fluorouracil. Viability data (B, C) are plotted as mean ± SEM of n = 3 or 4 technical replicates.
