# Supplementary material for: Nonsense‐mediated decay factor SMG7 sensitizes cells to TNFα‐induced apoptosis via CYLD tumor suppressor and the noncoding oncogene Pvt1
Source: Mol Oncol. 2020 Jul 13;14(10):2420–35. doi: 10.1002/1878-0261.12754 (PMC7530794; doi:10.1002/1878-0261.12754)
Supplement: Supplementary file 2 — Fig. S2. Gene Set Enrichment Analysis in Smg7−/− cells. [file MOL2-14-2420-s002.pdf]

	Gene Set Name	Description	*p*-value	FDR
**Upregulated**	MLL-AF4 fusion targets	Specific signature shared by cells expressing AF4-MLL alone and those expressing both AF4-MLL and MLL-AF4 fusion proteins.	2.61E-09	5.90E-05
	Matrisome	Ensemble of genes encoding extracellular matrix and extracellular matrix-associated proteins	8.65E-08	9.77E-04
	Prostate cancer	Genes up-regulated in prostate cancer samples from African-American patients compared to those from the European-American patients.	6.12E-07	4.61E-03
**Downregulated**	Apoptosis by serum deprivation	Genes up-regulated in ME-A cells (breast cancer) undergoing apoptosis upon serum starvation	9.47E-11	1.16E-06
	Stem cell	Genes up-regulated in freshly isolated CD31- (stromal stem cells from adipose tissue) versus the CD31+ (non-stem) counterparts.	5.18E-10	3.19E-06
	Stem cell cultured vs fresh	Genes up-regulated in cultured stromal stem cells from adipose tissue, compared to the freshly isolated cells.	2.14E-09	8.01E-06

Fig. S2: Gene Set Enrichment Analysis in *Smg7 -/-* cells.

Top identified clusters identified using top 100 up-/down-regulated genes in *Smg7 -/-* cells relative to parental cells detected in triplicate RNAseq samples.
