# Supplementary material for: Nonsense‐mediated decay factor SMG7 sensitizes cells to TNFα‐induced apoptosis via CYLD tumor suppressor and the noncoding oncogene Pvt1
Source: Mol Oncol. 2020 Jul 13;14(10):2420–35. doi: 10.1002/1878-0261.12754 (PMC7530794; doi:10.1002/1878-0261.12754)
Supplement: Supplementary file 4 — Fig. S4. Transcriptional changes in Smg7−/− cells. [file MOL2-14-2420-s004.pdf]

A

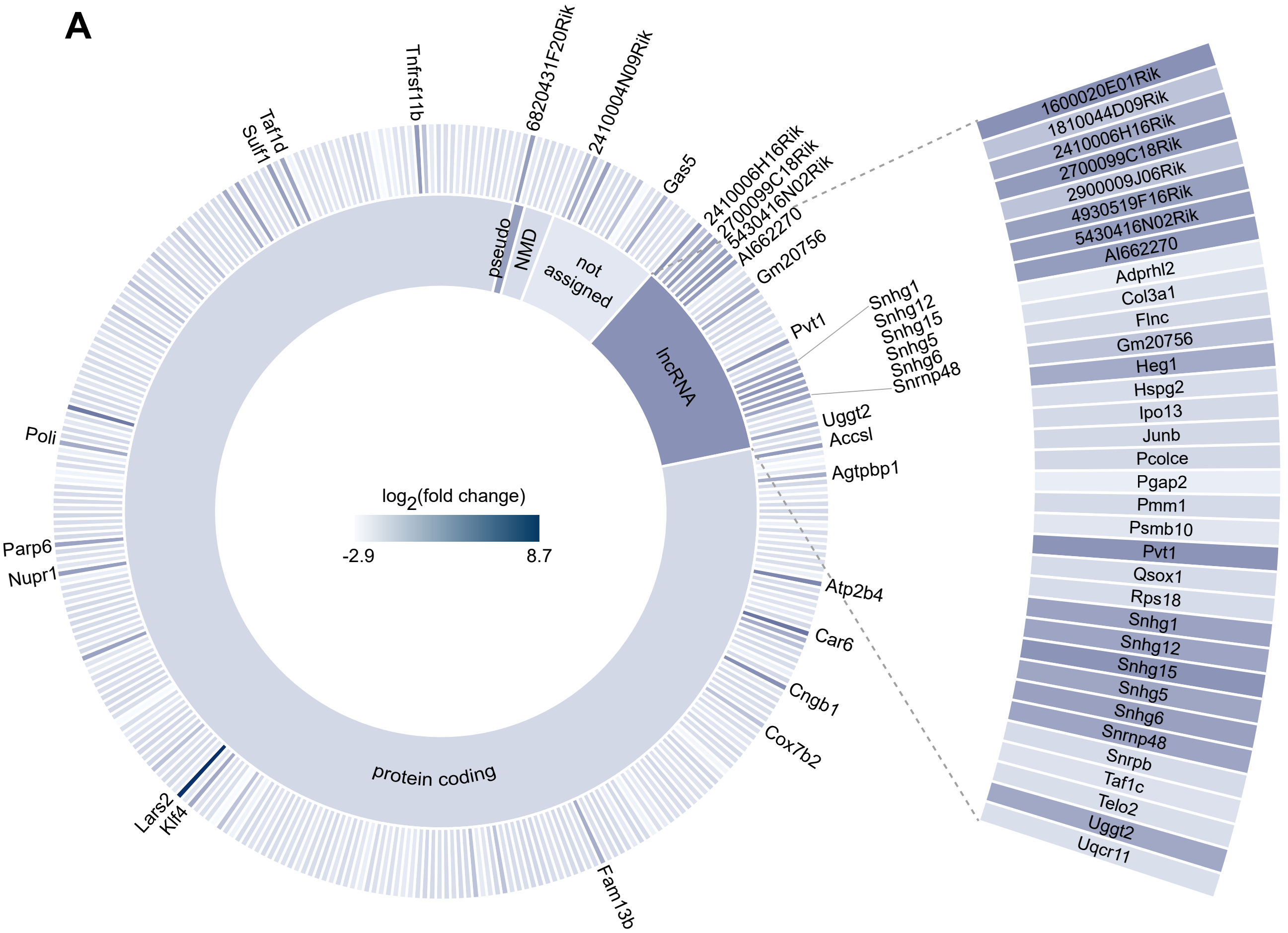

B

cell line	total mapped reads	multiple align (%)	total rate (%)
parental	9.6 x 10^6	23.9	7.3
Smg7-/-	13.4 x 10^6	67.9	13.5

C

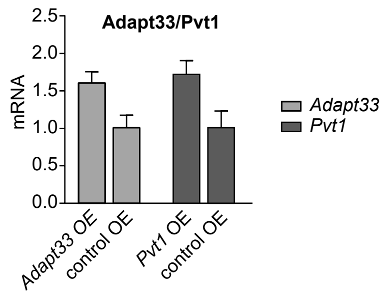

D

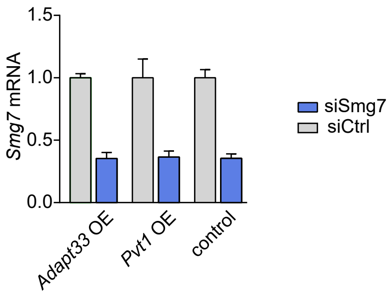

Fig. S4. Transcriptional changes in *Smg7 -/-* cells.

(A) Sunburst chart and indicated gene fold change in *Smg7 -/-* cells.

(B) Quantified mapped non-coding reads.

(C,D) qPCR validation of *Adapt33* and *Pvt1* mRNA and *Smg7* KD efficiency in *Adapt33* and *Pvt1* OE cells. Expression data are shown as mean ± SD of n = 3 technical replicates.
