# Supplementary material for: Nonsense‐mediated decay factor SMG7 sensitizes cells to TNFα‐induced apoptosis via CYLD tumor suppressor and the noncoding oncogene Pvt1
Source: Mol Oncol. 2020 Jul 13;14(10):2420–35. doi: 10.1002/1878-0261.12754 (PMC7530794; doi:10.1002/1878-0261.12754)
Supplement: Supplementary file 3 — Fig. S3. Quantitative mass spectrometry analysis of Smg7−/− compared to parental MF cells. [file MOL2-14-2420-s003.pdf]

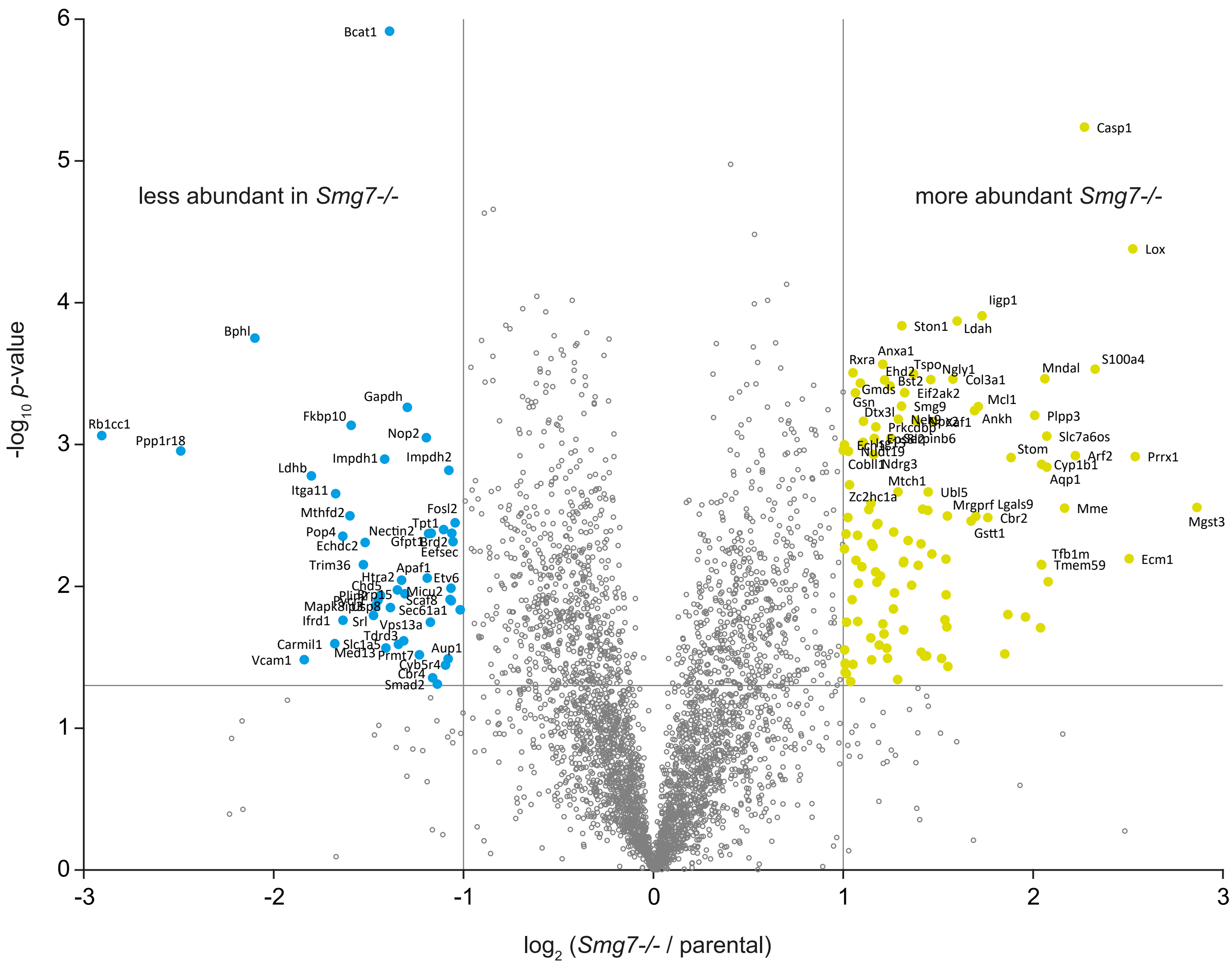

Fig. S3. Quantitative mass spectrometry analysis of *Smg7 -/-* compared to parental MF cells.
The $\log_2$ of the normalized protein abundance ratios *Smg7 -/-* / parental and $-\log_{10}$ of corresponding *p*-values of proteins quantified by at least two unique peptides from three replicate samples were plotted in a volcano plot. Proteins being significantly ($p < 0.05$) more abundant in *Smg7 -/-* are given in yellow and less abundant in *Smg7-/-* are given in blue (with a fold-change cut-off of 2-fold).
